# Improvement of Hydrodynamics-Based Gene Transfer of Nonviral DNA Targeted to Murine Hepatocytes

**DOI:** 10.1155/2013/928790

**Published:** 2013-03-17

**Authors:** Shingo Nakamura, Tadaaki Maehara, Satoshi Watanabe, Masayuki Ishihara, Masahiro Sato

**Affiliations:** ^1^Department of Surgery, National Defense Medical College, 3-2 Namiki, Saitama, Tokorozawa 359-8513, Japan; ^2^Division of Animal Science, National Institute of Agrobiological Sciences, 2 Ikenodai, Ibaraki, Tsukuba 305-0901, Japan; ^3^Research Institute, National Defense Medical College, 3-2 Namiki, Saitama, Tokorozawa 359-8513, Japan; ^4^Section of Gene Expression Regulation, Frontier Science Research Center, Kagoshima University, 1-21-40 Kohrimoto, Kagoshima, Kagoshima 890-0065, Japan

## Abstract

The liver is an important organ for supporting the life of an individual. Gene transfer toward this organ has been attempted in many laboratories to date; however, there have been few reports on improved liver-targeted gene delivery by using a nonviral vector. In this study, we examined the effect of various types of gene delivery carriers on enhancing the uptake and gene expression of exogenous DNA in murine hepatocytes when a hydrodynamics-based gene delivery (HGD) is performed via tail-vein injection. Mice were singly injected with a large amount of phosphate-buffered saline containing reporter plasmid DNA and/or with a gene delivery carrier. One day after the gene delivery, the animals' livers were dissected and subjected to biochemical, histochemical, and molecular biological analyses. The strongest signal from the reporter plasmid DNA was observed when the DNA was mixed with a polyethylenimine- (PEI-) based reagent. Coinjection with pCRTEIL (a *loxP*-floxed reporter construct) and pTR/NCre (a liver-specific Cre expression vector) resulted in the liver-specific recombination of pCRTEIL. The combination of PEI with HGD would thus be a valuable tool for liver-specific manipulation to examine the function of a gene of interest in the liver and for creating liver disease models.

## 1. Introduction

*In vivo* gene delivery has been widely used in various biotechnological fields as a valuable tool for elucidating gene function and for creating disease animal models. It is usually performed by a nonviral approach or a viral approach [1–3]. The former approach mainly depends on the use of plasmids, whereas the latter approach depends on the use of viral vectors such as the adenoassociated virus, retrovirus, and lentivirus. The nonviral approach has several advantages such as less toxicity and less immunogenicity, and this approach is safer and easier to prepare [4]. However, this approach has limited gene delivery efficiency and a short duration of transgene expression. 

The liver is a very important organ in an individual, and defects in the organ cause a serious threat to life. Therefore, scientists have focused on developing gene therapy that is targeted to hepatocytes and developing regenerative research for curing an injured liver [1, 5]. In animal experiments, the gene transfer efficiency in the liver after infection with adenoviral vectors is 80%; in plasmid-based gene delivery, it is only 10% to 15% [5, 6]. Liu and colleagues first developed a novel method to transfect hepatocytes with plasmid DNA *in vivo* with a relatively high degree of efficiency (approximately 40%) [7]. They employed a rapid intravenous injection of a large amount of solution containing naked plasmid DNA. This procedure is now called hydrodynamics-based gene delivery (HGD). This technology appears to be simple for the efficient transfection of hepatocytes, but unfortunately only a few trials to improve this technology have been made to date [8, 9]. Even with the HGD approach, it seems difficult to achieve a tissue-specific, continuous, and strong expression of a gene of interest (GOI). 

We previously demonstrated the usefulness of repeated* in vivo* gene delivery to elevate gene delivery efficiency. In this procedure, repeated intravenous injections of liposome-encapsulated plasmid DNA are administered to achieve a high degree of transgene expression in murine glomerular epithelial cells [10]. To enhance the gene expression of a GOI under the transcriptional control of a weak and tissue-specific promoter, we furthermore employed a Cre-*loxP* system and achieved approximately fourfold enhanced and tissue-specific expression of the target cDNA *in vivo *[11]. Through these experiments, *in vivo *gene delivery was performed via tail-vein injection of nonviral DNA that had been encapsulated by DMRIE-C (Invitrogen Co., Carlsbad, CA, USA), a liposome that the manufacturer has qualified as an* in vivo *DNA carrier. However, we believe that these previous approaches were inefficient on account of using the liver as a target organ for gene delivery since hepatocytes are frequently not transfected, compared with renal cells or other cells (unpublished data).

In this study, we attempted to improve HGD by testing several types of gene delivery carriers (including cationic lipid, polymers, and glycine reagents) to achieve a highly efficient gene delivery that is targeted to the murine liver. We furthermore tested whether the liver-specific expression of a GOI is possible when the aforementioned Cre-*loxP*-based system, which enables enhanced gene expression of a GOI, is under the transcriptional control of a weak, but liver-specific promoter.

## 2. Materials and Methods

### 2.1. Plasmid Vectors

**Figure 1:**
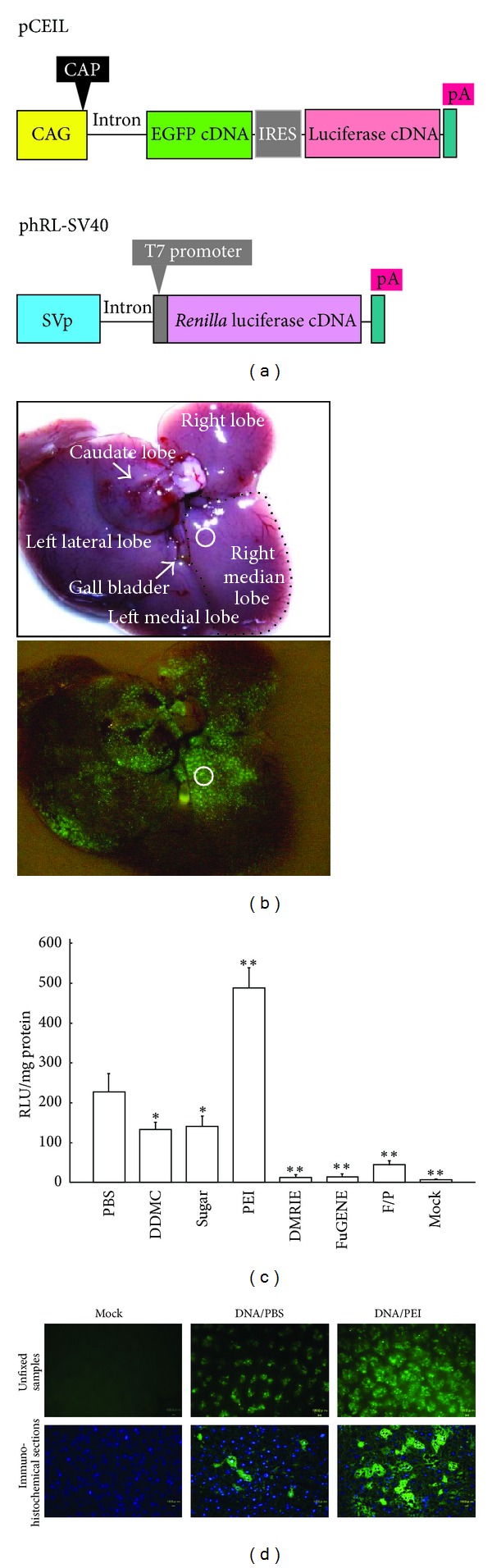
(a) The plasmid vectors, pCEIL and phRL-SV40, used for HGD are targeted to murine liver. (b) A liver dissected one day after the HGD of pCEIL DNA dissolved in PBS(-). The whole liver is photographed under light (upper panel) and under light and UV (lower panel). There is notable EGFP-derived green fluorescence in the region surrounding the large aorta. Particularly, the area in the right median lobe (enclosed by the circle) is extensively transfected. We therefore focused our study on this region. CAG: cytomegalovirus enhancer + chicken *β*-actin promoter; CAP site: transcription start site; EGFP: enhanced green fluorescent protein cDNA; IRES: internal ribosomal entry site; Pa: poly(A) sites; SVp: SV40 early promoter. (c) The measurement of luc activity in the liver one day after transfection with pCEIL/PBS(-) (PBS); pCEIL/PBS(-)/DDMC (DDMC); pCEIL/PBS(-)/SugarFect (Sugar); pCEIL/PBS(-)/*in vivo*-jet PEI (PEI); pCEIL/PBS(-)/DMRIE-C (DMRIE); pCEIL/PBS(-)/FuGENE HD (FuGENE); pCEIL/PBS(-)/F/P MPs (F/P); or PBS(-) alone (Mock). In each group, phRL-SV40 is included as the gene transfer control. The data (in relative light units (RLU) per milligram of protein) are presented as the mean ± the standard error (*n* = 6). For comparison of pCEIL/PBS(-) (PBS) and experimental groups, Scheffe's post hoc test was used with findings of *P* < 0.01 marked with double asterisk and *P* < 0.05 with an asterisk, respectively. (d) Fluorescence in the liver samples one day after transfection with PBS(-) alone (Mock); pCEIL/PBS(-) (DNA/PBS); or pCEIL/PBS(-)/*in vivo*-jet PEI (DNA/PEI). Unfixed liver samples of the right medial lobe (upper row panels) and immunohistochemical specimens (lower row panels) were examined with a fluorescence stereomicroscope to detect EGFP. Note the widespread bright cytoplasmic fluorescence in the liver sample after the introduction of pCEIL/PBS(-)/*in vivo*-jet PEI.

**Figure 2:**
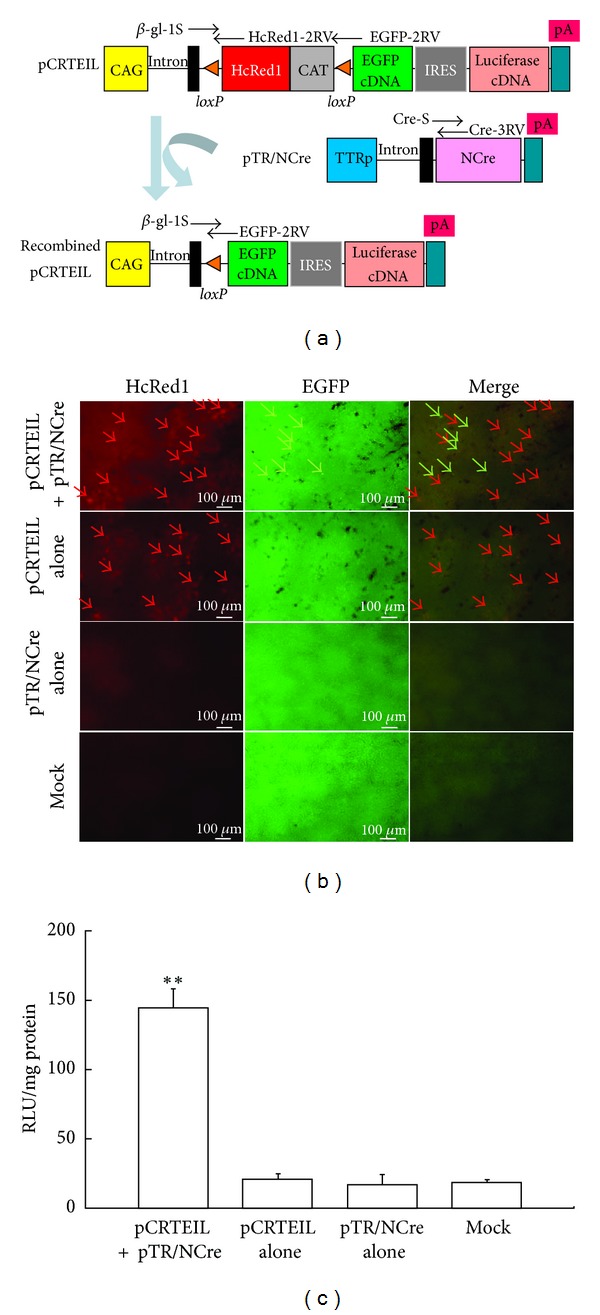
(a) The schematic for Cre-*loxP*-based recombination, using pCRTEIL as the reporter transgene. Before recombination, the floxed HcRed1/CAT hybrid sequence is expressed under the control of the CAG promoter, whereas the EGFP and luc cDNAs are silent. Cre-mediated recombination results in the deletion of the floxed sequence and the subsequent generation of recombined pCRTEIL. This induces the expression of the EGFP and luc cDNAs. CAG: cytomegalovirus enhancer + chicken *β*-actin promoter; CAT: chloramphenicol acetyltransferase gene; EGFP: enhanced green fluorescent protein cDNA; HcRed1: far-red fluorescent variant protein derived from the sea anemone *Heteractis crispa*; IRES: internal ribosomal entry site; pA: poly(A) sites; TTRp: mouse transthyretin promoter. (b) *In vivo* Cre-mediated recombination in pCRTEIL.Unfixed liver samples were dissected one day after HGD with pCRTEIL and pTR/NCre; pCRTEIL alone; pTR/NCre alone; or PBS(-) alone (Mock) and then were immediately inspected for HcRed1-derived red fluorescence (red arrows) or EGFP-derived green fluorescence (green arrows). Mixed fluorescence (indicated by green and red arrows in the “merge” column) is present only in the liver samples transfected with pCRTEIL and pTR/NCre. (c) Luc activity of the liver samples described in A. The data (in RLU per milligram of protein) are presented as the mean ± the standard error (*n* = 5). For comparison of PBS(-) alone (Mock) and the other groups, Scheffe's post hoc test was used with findings of *P* < 0.01 marked with double asterisk.

The pCEIL plasmid (Figure 1(a)) is a reporter plasmid driven by a chicken *β*-actin-based CAG promoter [12]. Two proteins (i.e., enhanced green fluorescent protein (EGFP) and firefly luciferase (luc)) are expected to be produced simultaneously from pCEIL since the internal ribosomal entry site (IRES) [13] permits translation of the two proteins from a single mRNA synthesis. The phRL-SV40 plasmid (Promega Co., Madison, WI, USA) (Figure 1(a)) was used as an internal control reporter, after cotransfecting it with pCEIL. The reporter plasmid pCRTEIL (Figure 2(a)) switches gene expression from red to green fluorescence when Cre expression occurs, as previously discussed [14]. The plasmid pTR/NCre (Figure 2(a)) enables the liver-specific expression of NCre (i.e., the Cre gene and the nuclear location signal) [15], the transcription of which is controlled by a liver-specific mouse transthyretin (i.e., prealbumin) promoter [16, 17].

### 2.2. Gene Delivery Carriers

We used the following reagents as gene delivery carriers: two cationic lipid reagents (DMRIE-C (Invitrogen Co., Carlsbad, CA, USA) and FuGENE HD (Promega Co.)); a polymer reagent (DDMC [Ryujyu Science, Aichi, Japan]); PEI reagent (*in vivo*-jet PEI (Polyplus-Transfection, Illkirch, France)), and two glycine reagents (SugarFect (Wako Pure Chemical Industries, Ltd., Osaka, Japan) and particles (F/P MPs)). The F/P MPs are nanoparticles (100–200 nm in diameter) that we have originally developed for the controlled delivery of recombinant angiogenic proteins [18–20]. They can be easily prepared by simple mixing of low-molecular-weight heparin with protamine, since these two molecules exhibit electrostatic interaction due to oppositely charged polyelectrolytes. 

### 2.3. *In Vivo* Gene Delivery by the Intravenous Injection of Plasmids

HGD was performed, as previously reported [7]. In brief, under sufficient anesthesia after intraperitoneal injection of sodium pentobarbital (Nembutal; Dainippon Sumitomo Pharma Co., Ltd., Tokyo, Japan), ICR male mice (five-week-old; CLEA Japan, Inc., Tokyo, Japan) were injected with a plasmid DNA-containing solution (one-tenth part of the weight/volume (in mL) per mouse, e.g., 3 mL/30 g of a mouse) by a syringe (3 mL luer lock type; Nipro, Inc., Osaka, Japan) fitted with a 27-gauge needle (Nipro). Injections were performed at a constant injection speed via tail vein and finished within 10 seconds. The injection was done by the same researcher in order to avoid artifactual effects in every experiment. The solution contained circular plasmid DNA dissolved in PBS(-) (i.e., phosphate-buffered saline without Ca^2+^ and Mg^2+^; pH 7.4) as the control or contained circular plasmid DNA mixed with various types of gene delivery carriers (as the experiment). In the latter solution, DNA was first mixed with each gene delivery carrier in accordance with the manufacturer-recommended protocols, and then the mixture was dissolved in PBS(-). For example, to evaluate *in vivo* transfection efficiency, we always introduced pCEIL (10 *μ*g) and phRL-SV40 (1 *μ*g) to each mouse. When we tested Cre-*loxP*-based gene switching *in vivo*, we mixed the following with 2.2 *μ*L of* in vivo*-jet PEI: phRL-SV40 (1 *μ*g) and pCRTEIL (10 *μ*g), pCRTEIL (5 *μ*g) and pTR/NCre (5 *μ*g), or pTR/NCre (10 *μ*g). The mixture was dissolved in PBS(-) before the intravenous injection. A mock injection of PBS(-) alone was performed as a control. Each group had six mice. 

All animal experiments were performed at the National Defense Medical College (Saitama, Japan) in accordance with the Guide for the Care and Use of Laboratory Animals. All efforts were made to minimize the number of animals used and to minimize their suffering.

### 2.4. Observation of Fluorescence

One day after HGD, the whole liver of a treated mouse was dissected and immediately transferred onto ice. The EGFP-derived fluorescence on the surface of a liver was then directly inspected and photographed under a fluorescence stereomicroscope (SMZ800; Nikon Co., Tokyo, Japan) with DM505 filters (BP460-490 and BA510IF; Nikon Co.). To detect fluorescence on a liver's internal area, it was cut in half by using a microtome blade (Feather Safety Razor Co., Ltd., Osaka, Japan) and then examined under a fluorescence microscope (BZ-8000; Keyence Co., Osaka, Japan). Some liver samples were fixed in 4% paraformaldehyde (PFA) in PBS(-) at 4°C for one day and then subjected to the standard immunohistochemical process [10] by using anti-GFP antibodies (1 : 100) (no. 598; Medical and Biological Laboratories Co., Ltd., Nagoya, Japan) and Alexa Fluor 488-conjugated goat anti-rabbit IgG antibodies (1 : 100) (Invitrogen Co.). After counterstaining with 4′,6-diamidino-2-phenylindole (DAPI), fluorescence in the immunohistochemical specimens was inspected under the BZ-8000 fluorescence microscope (Keyence Co.).

### 2.5. Assay for Luciferase Activity

One day after HGD, luc assay was performed by using a kit (Dual-Luciferase Reporter Assay System, #E1910; Promega Co.), as previously described [11]. Tissue protein determinations were performed by using Bradford reagent (Bio-Rad Laboratories, Inc., Hercules, CA, USA). Six liver samples were tested in each transfection group, and the values were presented as the means ± the standard deviation (SD). Statistical analysis was performed by repeated-measures analysis of variance (ANOVA). Scheffe's post hoc test was used for multiple comparison. *P* values were calculated using StatView 5.0 J for Windows (SAS Institute Inc., NC, USA).

### 2.6. Detection of Transgenes by Polymerase Chain Reaction

Genomic DNA from the transfected liver was isolated, as previously described [21]. Polymerase chain reaction (PCR) amplification reactions were performed, as previously described [11], in a total volume of 10 *μ*L reaction mixture with each of the following primer sets (Figure 2(a)): (1) *β*-gl-1S (5′-TGT GCT GTC TCA TCA TTT TGG-3′) and HcRed1-2RV (5′-CCT CGG TCA CGT GGA TTC TCA-3′) produce 260 bp fragments from the 5′ portion of the HcRed1 cDNA in pCRETIL;(2) Cre-S (5′-TTA CCG GTC GAT GCA ACG AGT-3′) and Cre-3RV (5′-CAT GTT TAG CTG GCC CAA ATG-3′) produce 262 bp fragments from the 5′ portion of the NCre gene in pTR/NCre; and (3) *β*-gl-1S and EGFP-2RV (5′-GTG CAG ATG AAC TTC AGG GTC-3′) yield 285 bp products corresponding to the recombined form of pCRTEIL. Five nanograms of pCRTEIL, pTR/NCre, and pCETD-12 [22] were used as the positive controls for detecting pCRTEIL, pTR/NCre, and the recombined form of pCRTEIL (a product after the Cre-mediated excision of pCRTEIL), respectively. The structure of pCETD-12 (consisting of CAG, *loxP*-flanked sequence containing EGFP cDNA and CAT gene, diphtheria toxin A-chain [DT-A] gene, and poly(A) sites) immediately upstream of EGFP cDNA is believed to be the same as the recombined form of pCRTEIL. Four microliters of each of the resulting PCR products were separated on a 2% agarose gel and they were stained with ethidium bromide (EtBr) for DNA visualization.

## 3. Results

### 3.1. Difference in Transgene Expression among the Hepatic Lobes

We first evaluated whether transgene expression varies among hepatic lobes after HGD. To test this, pCEIL and phRL-SV40 were intravenously administrated to the male mice. The use of the pCEIL plasmid for evaluating *in vivo* gene delivery efficiency is beneficial since it enables qualitative analysis (i.e., observation of EGFP-derived fluorescence under a fluorescence microscope) and quantitative analysis (i.e., biochemical measurement of luc activity) of the expression of the GOI. As Figure 1(b) (lower panel) shows, each lobe had transgene expression. The right median lobe exhibited highest degree of luc activity. When the luc activity in the right median lobe is defined as 100%, those in caudate lobe, left lateral lobe, left medial lobe, and right lobe were 89%, 83%, 50% and 48%, respectively. Notably, in the right median lobe, a strong and widespread EGFP expression was usually observed in the region surrounding the hepatic large aorta. Since EGFP fluorescence was always discernible in the right median lobe (indicated by the circle in Figure 1(b)) near the gall bladder, we focused on this area in our study. 

### 3.2. Improvement of Gene Delivery Efficiency by Employing Gene Delivery Carriers

We used several gene delivery carriers to test whether adding a commercially available gene delivery carrier to DNA dissolved in PBS(-) would increase the gene delivery efficiency in hepatocytes. A measurement of the luc activity demonstrated that transfection with pCEIL and phRL-SV40 mixed with *in vivo*-jet PEI yielded the highest degree of luc activity (Figure 1(c)). The luc activity was approximately 2.5-fold higher and significantly higher (*P* < 0.01) than its activity in a liver transfected with pCEIL and phRL-SV40 dissolved in PBS(-). The other gene delivery reagents, however, appeared to be ineffective for improving gene delivery efficiency targeted to hepatocytes. These findings were also confirmed by observing EGFP-derived fluorescence. Direct inspection for fluorescence by using unfixed liver specimens demonstrated that transfection with pCEIL and phRL-SV40 mixed with *in vivo*-jet PEI yielded a high degree of fluorescence in the liver (Figure 1(d), upper panel of the “DNA/PEI” column). In the immunohistochemical specimens of the transfected liver, fluorescence was mainly localized in the cytoplasm of the hepatocytes (Figure 1(d), lower panel of the “DNA/PEI” column). Based on these results, we decided to employ *in vivo*-jet PEI with HGD as the gene delivery carrier for efficient *in vivo* gene delivery toward hepatocytes. 

### 3.3. Cre-*loxP*-Based Gene Switching in a Liver-Specific Manner

HGD-mediated gene transfer is not liver-specific, but liver is an organ predominantly transfected with this method, compared to the other major organs [7]. Our final goal was to manipulate hepatocytes *in vivo* through gene products produced from exogenous DNA such as plasmid constructs. This always requires the liver-specific expression of a GOI. To realize this system, we used a Cre-*loxP*-mediated system, which is termed CRTEIL [14]. As Figure 2(a) shows, when pCRTEIL is cointroduced with pTR/NCre and both plasmids are simultaneously taken up by the same hepatocyte, then Cre-mediated excision occurs. This event leads to the generation of recombined pCRTEIL and subsequently switches the gene expression from red to green. If Cre-mediated excision does not occur in cells carrying pCRTEIL, these cells only express HcRed1-derived red fluorescence. One day after the HGD of pCRTEIL and TR/NCre with *in vivo*-jet PEI, the mice's livers, kidneys, and lungs were dissected and immediately inspected for fluorescence. HcRed1-derived red fluorescence and EGFP-derived green fluorescence were observed in the liver when pCRTEIL and pTR/NCre were coinjected (the “pCRTEIL+pTR/NCre” row in Figure 2(b)). As we expected, red fluorescence (but not green fluorescence) was observed in the livers injected with pCRTEIL alone (the “pCRTEIL alone” row in Figure 2(b)). No fluorescence was noted in the livers injected with pTR/NCre alone and in the mock-injected livers (the rows labeled “pTR/NCre alone” and “Mock,” resp., in Figure 2(b)). In the lungs and kidneys, the restricted and weak expression of red fluorescence was present, but the organs had no noticeable expression of green fluorescence (data not shown). These findings suggest that a liver-specific recombination of pCRTEIL occurred, although pCRTEIL is distributed in the liver, the lungs, and the kidneys. Measurement of luc activity confirmed the elevated expression of the GOI (i.e., EGFP and luc) in the liver samples that had been coinjected with pCRTEIL and pTR/NCre (Figure 2(c)). 

**Figure 3:**
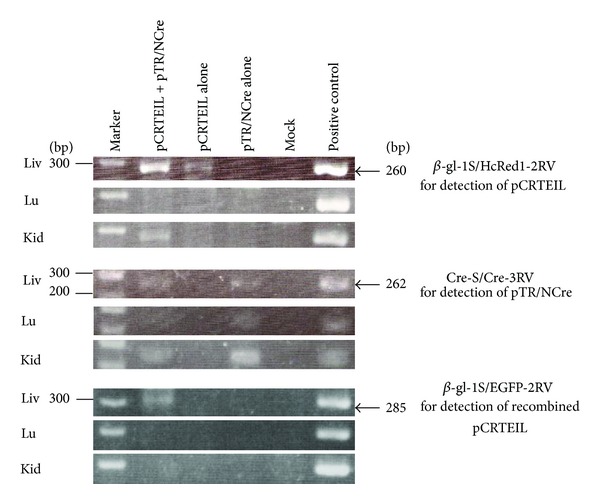
PCR analysis of the transgene for the identification of the recombined form of pCRTEIL in the liver. Through HGD, the mice received an intravenous injection of pCRTEIL and pTR/NCre; pCRTEIL alone; pTR/NCre alone; or PBS(-) alone (Mock). One day after HGD, the livers (Liv), lungs (Lu) and kidneys (Kid) of the mice in each group were dissected to obtain the transgene. PCR was performed by using the primer set described previously in the Materials and Methods section.

PCR analysis was performed to confirm the liver-specific Cre-mediated recombination event at a molecular level. PCR using the primer set *β*-gl-1S and HcRe1-2RV to detect pCRTEIL revealed that the amplified products (260 bp) were in the livers injected with pCRTEIL and pTR/NCre or injected with pCRTEIL alone, but not in the livers injected with pTR/NCre alone (Figure 3, upper three panels). The presence of pTR/NCre was similarly confirmed by using the primer set Cre-S and Cre-3RV, which detected pTR/NCre in livers injected with pCRTEIL and pTR/NCre or with pTR/NCre alone, but not in livers injected with pCRTEIL alone (Figure 3, middle three panels). When using the primer set *β*-gl-1S and EGFP-2RV to identify the recombined form of pCRTEIL, only livers injected with pCRTEIL and pTR/NCre had the PCR-amplified products (285 bp), as expected (Figure 3, lower three panels). 

## 4. Discussion 

On performing tail-vein-mediated* in vivo* gene delivery, it is important to maintain the stability of the introduced DNA in the blood stream. It has been reported that naked DNA administrated into blood vessels is indeed amenable to degradation by DNase that is present in the blood [23]. A previous report on HGD adopted naked plasmid DNA for the transfection of hepatocytes, but achieved a relatively high degree of gene delivery efficiency [7]. This is probably because a large volume of DNA solution can dilute all of the components in blood, including DNase, which will allow the exogenous naked DNA to be easily incorporated intact inside a cell. On the other hand, a naked DNA complexed with DNA carriers is sometimes used to enhance the stability of the DNA against DNase degradation [24]. Therefore, it is reasonable to believe that DNA mixed with a gene delivery carrier that is introduced through HGD would greatly increase the transfection efficiency of hepatocytes, compared with the introduction of naked DNA alone. In fact, pCEIL DNA mixed with* in vivo*-jet PEI yielded approximately 2.5-fold higher (*P* < 0.01) luc activity than did naked DNA dissolved in PBS(-) (Figure 1(c)). 

The gene delivery carriers we examined, except for *in vivo*-jet PEI, were less effective for improving transfection efficiency in the liver. What is the difference in the transfection efficiency between* in vivo*-jet PEI and the other reagents? 

High blood pressure caused by HGD enlarges the hepatocyte membrane pores (to approximately 100 nm in diameter) [25]. This facilitates the passage of exogenous DNA across the liver capillary endothelial cells and taken up by hepatocytes [8]. Based on this point of view, it seems that the size of a complex formed between DNA and a gene delivery carrier may affect the transfection efficiency. It has been reported that the size of DNA/DMRIE-C is 150 nm [26]; DNA/SugarFect, 130 nm (protocol; MedGEL Co., Ltd, Kyoto, Japan); DNA/F/P MPs, 150 nm (unpublished data); or DNA/*in vivo*-jet PEI, 80 nm [27]. Evaluation of particle size by using a light microscope revealed that the size of *in vivo*-jet PEI/DNA complex was 100 nm or less in size (data not shown). Therefore, the size of DNA/*in vivo*-jet PEI is smaller than the membrane pores of hepatocytes enlarged by HGD. The background data may support using DNA mixed with *in vivo*-jet PEI to achieve efficient hepatocyte transfection.

Administration of DNA/DDMC, DNA/SugarFect, or DNA/F/P MPs complexes resulted in transfection of hepatocytes; its efficiency was relatively low (Figure 1(c)). This event might have been promoted via phagocytosis of the DNA complex that had survived under DNase-rich blood stream and attached to the hepatocyte's membrane, as previously suggested [28]. Particularly, polymer carrier (DDMC) has strong anti-DNase activity [29]. Furthermore, liver-specific gene delivery via asialoglycoprotein receptor was also reported [30, 31]. Gene delivery using carbohydrates (such as SugarFect and/F/P MPs) as a gene delivery carrier may employ such asialoglycoprotein receptor-mediated gene delivery system. 

Notably, introduction of DNA encapsulated by lipid reagents such as DMRIE-C and FuGENE HD failed to transfect hepatocytes efficiently (Figure 1(c)). The most probable cause for this failure may be due to the inability toliberate plasmid DNA from the DNA-lipid complex when the complex is incorporated by a cell, since there are a few enzymes involved in release of plasmid DNA from a DNA-lipid complex in liver [32]. 

Among the several gene delivery carriers tested, we successfully achieved a relatively high *in vivo* gene delivery rate in hepatocytes by employing HGD and* in vivo*-jet PEI as the gene delivery carrier. The following reasons may explain this: (1) the size of DNA/*in vivo*-jet PEI complex is relatively small (approximately 80 nm), which facilitates the physical incorporation of the complex by hepatocytes under a high degree of blood pressure; (2) the DNA complex with PEI is resistant against blood DNase [33]; (3) a complex that is not transferred into the hepatocytes by HGD may be taken up (i.e., separately from the HGD process) via phagocytosis of the DNA complex attached to the cell surface; and (4) the DNA complex incorporated by a hepatocyte may be rapidly dissociated by the proton sponge mechanism [33], which elevates the expression of the exogenous DNA in a transduced cell. 

Kang and colleagues recently demonstrated the successful liver-targeted siRNA delivery by a hydrodynamics-based injection of DNA complexed with a PEI-pullulan carrier [34]. They report that, although an HDS-based injection of DNA complexed with PEI frequently causes the death of mice and decreases the expression of siRNA, the addition of pullulan to the DNA/PEI complex prevents the frequent deaths of the mice. Their findings appear inconsistent with the findings of our present study indicating that the combined use of HGD and PEI is beneficial for the efficient expression of a GOI. We furthermore did not experience the sudden deaths of mice after HGD. One reason may be that Kang and colleagues used commercially available PEI, whereas we used *in vivo*-jet PEI, which the manufacturer has formulated as a less toxic *in vivo* gene delivery reagent. In general, PEI itself is known to be toxic and induce cell death. However, *in vivo*-jet PEI does not induce any significant inflammatory response caused by TNF-alpha, IL-6, and IL-12/IL-23 [35]. Actually, in our preliminary experiment, no pathological abnormality was noted in the H-E-stained liver specimens obtained after transfection with DNA/*in vivo*-jet PEI complex (data not shown). Notably, Kang et al. [34] reported frequent death of individuals after HGD which may depend on mouse strain used. We have often gotten to witness an astonishing difference in results depending on mouse strain (data not shown). In this context, it would be required to explore optimal condition for achieving best gene delivery performance with reducing accidental individual death as possible, and we are planning to examine which mouse strain, its sex or its age, would be suitable for this purpose. On the other hand, it would be of interest to test whether HDS-based injection of DNA/*in vivo*-jet PEI/pullulan can improve our liver-targeted transfection efficiency and possibly avoid the previously mentioned safety problems, because pullulan has been used for liver targeting since it exhibits high affinity for the asialoglycoprotein receptor in the liver [36]. 

By using a combination of HGD and *in vivo*-jet PEI as the gene delivery carrier, a researcher can manipulate the function of hepatocytes by overexpressing a GOI at the time the researcher desires. The promoter activity of a tissue-specific promoter is generally believed to be weak, compared with activity of a ubiquitous and strong promoter such as the chicken *β*-actin-based promoter CAG [12]. To achieve a system allowing the transgene to be expressed strongly in a tissue-specific manner, we have developed a Cre-*loxP*-based system (i.e., enhanced tissue-specific gene expression (ETSGE)) that enables enhanced transgene gene expression under a weak tissue-specific promoter [11]. This system is simple. It can be performed by cotransfection of a tester plasmid (comprising CAG, *loxP*-flanked stopper sequence, GOI, and poly(A) sites) and a plasmid that confers Cre gene expression under a tissue-specific promoter. The tissue- or cell-specific expression of a GOI occurs in a tissue or cell carrying a tester and Cre-expressing plasmids. In this study, we confirmed that coupling HGD with *in vivo*-jet PEI is useful for realizing the liver-specific and strong expression of a GOI (in this case, EGFP and luc) (Figures 2(b) and 2(c)). Our future subject using this system is to create fibrosis models in mice by hepatocyte-specific expression of DT-A gene. We already constructed a tester plasmid pCETD-12 [22]. Coinjection with pCETD-12 and pTR/NCre would cause massive hepatocyte death, leading to generation of liver fibrosis.

## 5. Conclusion 

Hydrodynamics-based nonviral gene delivery toward hepatic cells is greatly improved when plasmid DNA is mixed with a polyethylenimine-based reagent. With this improved technique, we succeeded in performing Cre-*loxP*-mediated gene switching in liver and showing lines of direct evidence for liver-specific transgene expression by liver-specific promoter. To our knowledge, these formulations are novel and in the future would be useful for gene-based manipulation of the liver. 

## Supplementary Material

The results of other transfection reagents were displayed as supplementary figure.
