# Supplementary figures and images for: Improvement of Hydrodynamics-Based Gene Transfer of Nonviral DNA Targeted to Murine Hepatocytes
Source: Biomed Res Int. 2013 Mar 17;2013:928790. doi: 10.1155/2013/928790 (PMC3613052; doi:10.1155/2013/928790)

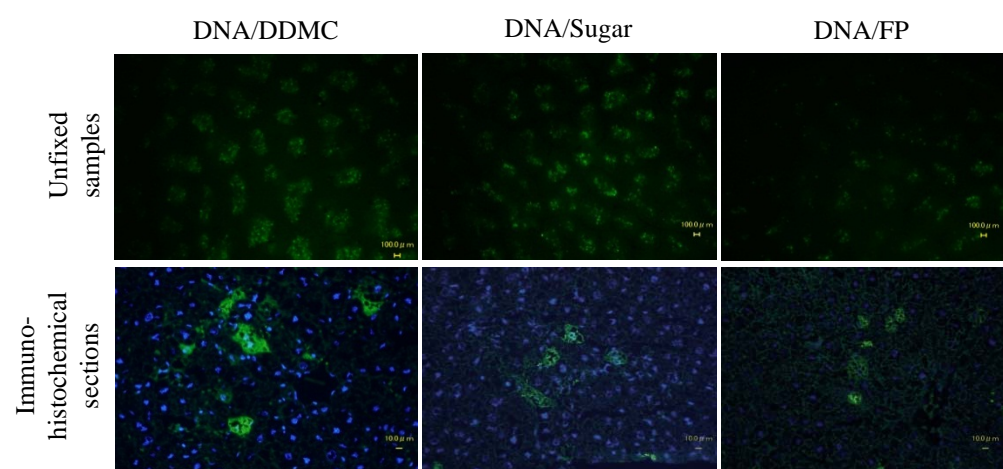

Supplementary Fig. 1D

Supplement: Supplementary file 1 — The results of other transfection reagents were displayed as supplementary figure. [file 928790.f1.pdf]
